# The Role of Viral Population Diversity in Adaptation of Bovine Coronavirus to New Host Environments

**DOI:** 10.1371/journal.pone.0052752

**Published:** 2013-01-07

**Authors:** Monica K. Borucki, Jonathan E. Allen, Haiyin Chen-Harris, Adam Zemla, Gilda Vanier, Shalini Mabery, Clinton Torres, Pamela Hullinger, Tom Slezak

**Affiliations:** Lawrence Livermore National Laboratory, Livermore, California, United States of America; Blood Systems Research Institute, United States of America

## Abstract

The high mutation rate of RNA viruses enables a diverse genetic population of viral genotypes to exist within a single infected host. In-host genetic diversity could better position the virus population to respond and adapt to a diverse array of selective pressures such as host-switching events. Multiple new coronaviruses, including SARS, have been identified in human samples just within the last ten years, demonstrating the potential of coronaviruses as emergent human pathogens. Deep sequencing was used to characterize genomic changes in coronavirus quasispecies during simulated host-switching. Three bovine nasal samples infected with bovine coronavirus were used to infect human and bovine macrophage and lung cell lines. The virus reproduced relatively well in macrophages, but the lung cell lines were not infected efficiently enough to allow passage of non lab-adapted samples. Approximately 12 kb of the genome was amplified before and after passage and sequenced at average coverages of nearly 950×(454 sequencing) and 38,000×(Illumina). The consensus sequence of many of the passaged samples had a 12 nucleotide insert in the consensus sequence of the spike gene, and multiple point mutations were associated with the presence of the insert. Deep sequencing revealed that the insert was present but very rare in the unpassaged samples and could quickly shift to dominate the population when placed in a different environment. The insert coded for three arginine residues, occurred in a region associated with fusion entry into host cells, and may allow infection of new cell types via heparin sulfate binding. Analysis of the deep sequencing data indicated that two distinct genotypes circulated at different frequency levels in each sample, and support the hypothesis that the mutations present in passaged strains were “selected” from a pre-existing pool rather than through de novo mutation and subsequent population fixation.

## Introduction

Three quarters of the recently discovered human pathogens are viral, and most of those are RNA viruses [1]. Some of these emergent viruses, such as HIV and SARS coronavirus (SARS-CoV), are capable of causing epidemics of human disease. RNA virus populations sustain high genetic diversity due to the low fidelity of their polymerase, short genome, high replication rates and large population size [2]. For this reason a single RNA virus population can consist of a multiplicity of slightly different genomes, sometimes referred to as a mutant spectra [3]. The high mutation rate of RNA viruses increases the ability of these viruses to adapt to diverse hosts (interspecies transmission events) and the potential cause new human and zoonotic diseases [4], however, very little is known about the particular mutations that enable interspecies transmission events to occur.

Coronaviruses are particularly adept at adapting to new hosts due in part to their amazing capacity for genome recombination. Coronaviruses have the largest genome of RNA viruses, consisting of 27–30 kb positive sense single-stranded RNA. Although recombination can lead to an interspecies transmission event, as was believed to be the case with SARS-CoV, accumulation of point mutations may also enable the coronaviruses to adapt to new host species [5]–[7].

The *Coronaviridae* subfamily *Coronovirinae* is composed of three genera based on serologic and genetic characteristics: *Alphacoronavirus* (formerly Group 1) includes viruses that infect pigs, dogs, cats and humans; *Betacoronavirus* (formerly Group 2) includes bovine, bat, human, horse, pig, rodent, and bat viruses; and *Gammacoronavirus* (formerly Group 3) which consists of viruses adapted to birds [7], [8]. Bovine coronavirus (BCoV) is a betacoronavirus which is related to SARS-CoV and has caused disease in humans on at least one occasion [9]. BCoV is known to use 9-*O-*acetylated sialic acid to bind to host cells, although a second receptor may be involved [10]. The spike protein which is present on the surface of the virion, determines host range and tissue tropism of coronvaviruses. The receptor binding domain of BCoV has not been determined [11], but a recent study by Peng at al. (2011) indicates that it falls within the N terminal domain [12]. BCoV infection may cause acute and severe diarrhea and respiratory symptoms in cattle especially under stressful conditions such as transport, but subclinical infections may occur in healthy cattle. BCoV isolates are generally obtained by inoculating nasal or fecal samples from infected cattle in human rectal tumor cells (HRT-18 cells), however the genetic changes in the viral population that allow this bovine virus to adapt to human cell lines have yet to be defined [13].

We are interested in understanding the role of natural viral population diversity in the adaptation of BCoV to new host environments; in particular, cell types that may enable the virus to spread via the respiratory route or to cause systemic disease. The mutational dynamics of BCoV was determined by serial passage of BCoV nasal samples in human and bovine lung and macrophage cell lines as well as human enteric cells (A549, EBL, THP-1, Bomac, and HRT-18, respectively). The consensus and subconsensus (variant) nucleotide sequences of cell culture passaged samples were compared to that of the natural “unpassaged” viral populations. The genome regions analyzed included nsp1and nsp3 (genes involved with evasion of the host innate immune response and other functions such viral replication), nsp14 (designated nsp11 in NCBI BCoV Reference Sequence: NC_003045.1; involved with polymerase fidelity), and the spike protein gene (determinant of host range) [7], [14]–[19]. Deep sequencing was used to obtain an accurate picture of viral population diversity before and after passage in cell culture.

## Results

### Virus Passage Experiments

Nasal samples were obtained from calves and tested for BCoV RNA via RT-PCR. Naturally-infected BCoV samples are often difficult to adapt to growth in cell culture [20], however, it is essential to use naturally-infected samples rather than laboratory adapted virus strains because isolation in cell culture is likely to result in adaptive mutations not present in the naturally circulating virus. Therefore, every step of the virus passage protocol was optimized to allow for the greatest chance of virus particle recovery before and during cell culture passage including incubation time of the virus on cells, RNA extraction protocol, and RT-PCR protocol. Although trypsin has been shown to enhance infection of HRT-18 cells [20], [21], it was not used in the infection protocol in order to keep protocols identical for infection of enteric, lung and macrophage cell lines. The three nasal samples (#’s 1, 27, and 59) that had the highest titer as determined by TaqMan RT-PCR were tested using the optimized protocols. A laboratory adapted strain, Nebraska (NEB) was included as a positive control for cell line infectivity.

Infection by the natural BCoV nasal samples was productive in only three of the five cell lines: THP-1, Bomac and HRT-18, as determined by RT-PCR, compared to all five cell lines productively infected by the lab adapted strain, NEB, as determined by RT-PCR. Viral growth was sustained through five passages in the THP-1 and HRT-18 cells and for four passages in the Bomacs (no viral RNA was detected by Taqman assay from passage 5 in Bomacs for any of the viruses except for the NEB strain). For each sample, viral RNA was extracted from samples prior to any passage (“unpassaged virus”) and from the first and the last passage experiments – passage 4 for the Bomacs and passage 5 for the THP-1 and HRT-18 cells.

### Viral Genome Amplification

Approximately 12 kilobase (kb) of the BCoV 30 kb genome was amplified using 16 primer sets designed to conserved regions of the genome (Table S1). The primer sets were used to amplify unpassaged and passaged viral RNA, however, in some cases not all primer sets yielded PCR product suggesting that changes occurred in the primer binding region during passage.

### Analysis of Consensus Sequence Data

**Figure 1:**
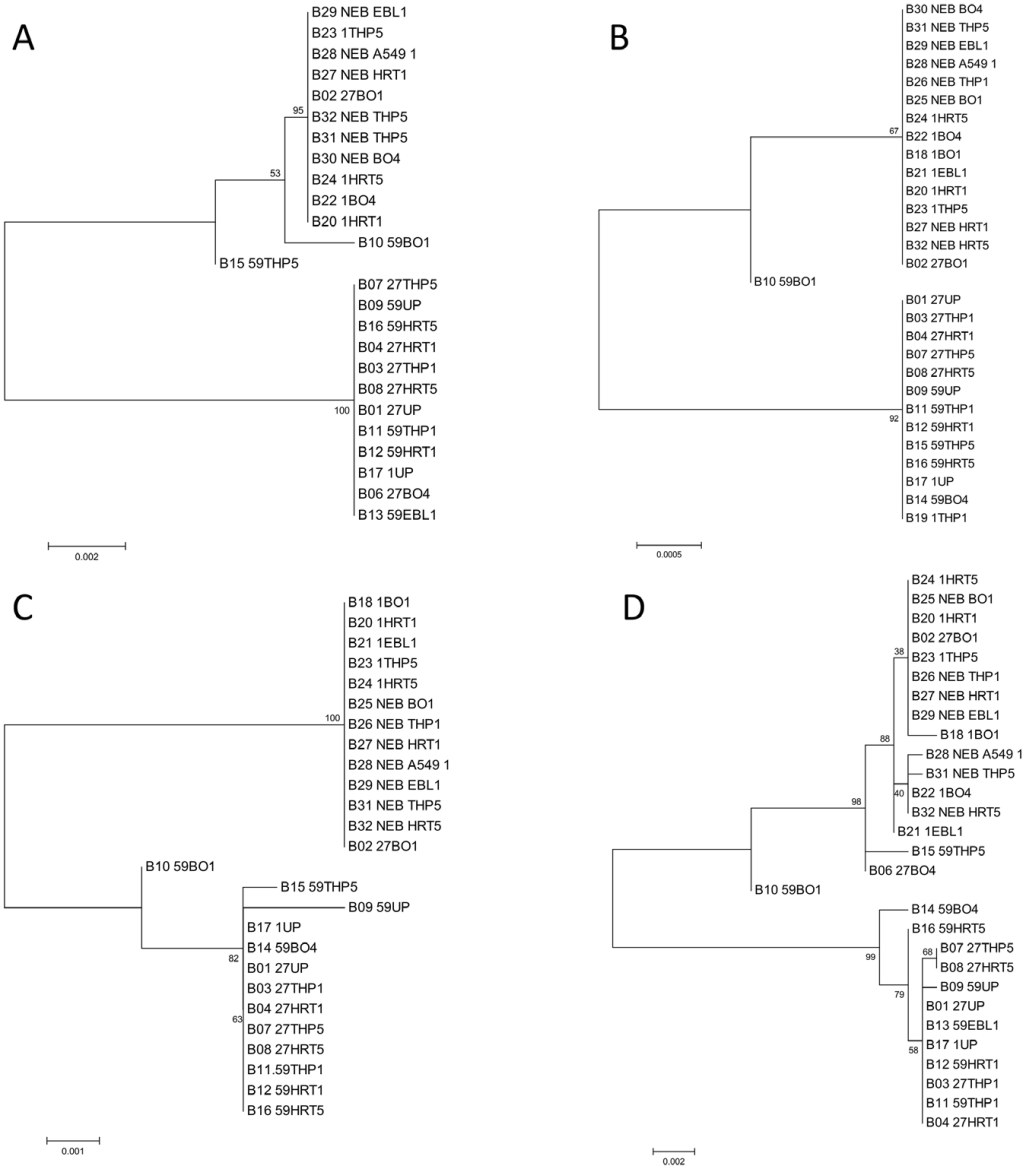
Phylograms generated using consensus sequence for the coding regions of Nsp1, Nsp3, Nsp14, and Spike. (A–D, respectively.) Samples are labeled using sample sequence identifier (B##), samples #, cell type, and passage number. Phylogeny was inferred by using the Maximum Likelihood method based on the Tamura-Nei model [42]. The tree with the highest log likelihood is shown. The percentage of trees in which the associated taxa clustered together is shown next to the branches. The tree is drawn to scale, with branch lengths measured in the number of substitutions per site. Codon positions included were 1st+2nd+3rd+Noncoding. All positions containing gaps and missing data were eliminated. There were a total of 1671 (nsp1), 851 (nsp3), 1561 (nsp14), and 1457 (spike) positions in the final dataset. The genomic regions included in the analysis span nts 2764–8460 (nsp1), 10858–11714 (nsp3), 17910–19472 (nsp14), and 23641–27732 (spike), as numbered according to accession AB354579. Analyses were conducted using MEGA5 [43]. Samples may be absent from analysis due to inadequate sequence data generated for a particular coding region, however in cases where fewer positions gave the same phylogram as that generated using more positions, fewer positions were used to allow inclusion of more samples in the phylogram.

The nsp1, nsp3, nsp14, and spike protein gene consensus sequences of passaged and unpassaged viruses were compared to identify any changes characteristic of serial passage. Phylogenetic analysis of the consensus sequence showed that two of the naturally-infected nasal samples (#’s 27 and 59) changed much more gradually compared to that of the third such sample (#1) which rapidly changed to resemble that of the laboratory adapted NEB strain after just one passage in cell culture, regardless of cell type (Figure 1). The samples formed two major clusters, one which included all the unpassaged samples (“UP” group) and one that included all the NEB reference strain samples and all the passaged samples derived from sample 1 (Passaged group, “P”). Regions of the genome varied in the number of observed mutations in the consensus sequence with the spike and nsp1 genes showing the most mutations due to passage. Interestingly, the consensus sequence of the viruses passaged in Bomac cells changed more rapidly as compared to that of the genomes of viruses passaged in other cell lines (Figure 1).

Most surprisingly, after passage in cell culture, the consensus sequences of many viral samples acquired a 12-nucleotide (nt) insert encoding 4 amino acids (Ser, Arg, Arg, Arg) located at nt 2737 of the spike gene, whereas none of the unpassaged samples contained this insert at the consensus level. This is illustrated in the phylogram of the spike protein (Figure 1D) where samples with the insert generally group together in the upper clade of the tree, although other point mutations differed between the groups as well. The four-amino acid insert was located immediately adjacent to the proteolytic cleavage site of the spike protein, and in the S2 subunit of the spike protein which is responsible for fusion entry of the virus into the host cell [22]. The addition of multiple basic residues to this region creates a furin cleavage site that may serve as an alternative to trypsin-dependent cleavage of the spike protein [23]. Data from the 454 sequencing (mean 931×coverage) showed the insert was also present at subconsensus level in some of the cell-passaged samples (Table 1). An in-depth analysis using Illumina ultra-deep sequencing (mean 37,901×coverage) showed that the insert was present at very low numbers in unpassaged samples but was sometimes enriched during passage in cell culture indicating a possible role in cell culture adaptation.

**Table 1:** Percentage of 454 reads containing the multibasic insert (Illumina data in parentheses).

Sample	27	59	1	NEB
UP	0.1(0.05)	0(0.01)	3(0.6)	na
BO.P1	87	37	92	88
BO.P4	32	(94)	94	95
THP.P1	0.07	13	93	88
THP.P5	0	20	88	94
HRT.P1	0	0.6	86	86
HRT.P5	0	21	88	88
EBL.P1	na	na	85	87
A549.P1	na	na	na	87

Columns are for the different samples: 27, 59, 1, and Nebraska strain. Samples are labeled using cell type, and passage number. na: sample data not available.

To determine if the insert sequence was present in the NEB sample that we obtained from NVSL before it was propagated in our laboratory, a second sample of this strain was requested from NVSL. Unfortunately this strain was no longer available due to a gap in production testing so a sample of the RNA from the NVSL NEB stock was obtained for testing purposes. This RNA sample was tested using a Taqman PCR assay designed to detect the insert and data showed that the insert was present at approximately the consensus level (>50%) in the RNA from NVSL (Table S2).

The insert reached consensus in all passages of nasal sample #1, passage 1 of sample #27 and passage 4 of sample #59 in Bomacs, but in all other sample-passage combinations it was present at subconsensus level between 0.01 and 37% (Table 1). This 12 nt insert was not present in any BCoV sequences available in GenBank (the NEB strain sequence is not available in GenBank). Because GenBank data typically reflect the consensus sequence present in a sample, it is possible that the insert is present at subconsensus level in other common lab-adapted strains of BCoV. To address this question, BCoV strain Mebus was obtained from BEI Resources and tested for presence of the insert using the Taqman PCR assay. No insert was detected in diluted or undiluted Mebus RNA, even though Mebus polymerase was present at approximately 13,000 copies per ng RNA.

Four additional bovine nasal samples that tested positive for the presence of BCoV polymerase by Taqman assay were tested for the presence of the insert using the Taqman assay. The polymerase gene was detected at very low levels in all of the samples (range 13–367 copies per 5 µl cDNA) and the insert was detected at very low levels in one of these unpassaged samples. Additionally, Illumina and 454 deep sequencing data had been generated from an unpassaged BCoV nasal sample, #74, for a previous study and these data were examined and found to have the insert present at a low level.

To verify the reproducibility of the rapid enrichment of the insert to consensus level in nasal sample #1, we used the Taqman insert assay to test for the presence of the insert in a second sample of #1 that was passed once in Bomac cells and was available from a previous experiment. The Taqman assay detected the insert in the sample in approximately the same numbers as the polymerase gene, thus indicating that the enrichment process is reproducible.

### Summary of Subconsensus Point Mutations

The number of viral genomic variants (nucleotide polymorphism occurring at subconsensus level) varied widely between samples (Table S3). Figure S1 shows that there is little correlation between variant count and coverage, suggesting that the wide differences in variant count could not be explained by variation in coverage by 454 sequencing.

To increase the sensitivity of variant detection, the three unpassaged samples were sequenced a second time using Illumina GA IIx sequencing (sample 59.BO.4 was also resequenced as its 454 data disagreed with Taqman results). Compared to 454 data, Illumina sequencing data detected substantially more variants in nasal sample #27 (81 to 226) and nasal sample #59 (10 to 122) (Table S3). Illumina data for nasal sample #1 showed only a modest increase of variant counts (from 207 to 260) compared to the 454 data. This is consistent with the fact that the majority of variants in nasal sample #1 occurred at higher frequency – high enough that most were detected with 454 sequencing.

To measure the abundance of a variant at a given position in the genome within a sample, we defined the frequency of a variant as the percentage of mapped reads that overlap the query position and contain the target variant divided by the total number of mapped reads that overlap the query position and make a base call of any type. The median variant frequency for samples #27 and #59 was 0.5% but 6% for sample #1. Thus, the increased variant count obtained from Illumina in samples #27 and #59 reflected the presence of a larger pool of variants present at lower frequency levels. On average, the fraction of the sequenced regions of the genome containing a variant was 0.5% for 454 data and 1.5% for the Illumina data (analysis restricted to bases where a consensus nucleotide is called). This is consistent with the higher coverage and sensitivity associated with our Illumina data.

### Variants in Unpassaged Samples become Consensus in Passaged Samples

**Figure 2:**
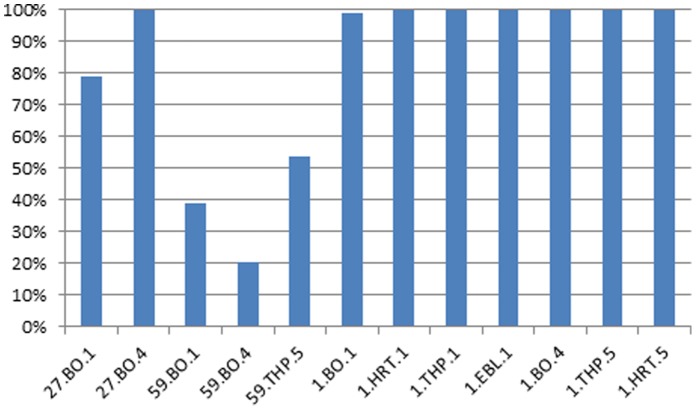
Percent of consensus SNPs that occur as subconsensus variants in unpassaged samples. The consensus sequence for each unpassaged parent sample was compared to its passaged descendants. For each sample that clustered away from the unpassaged parent (Figure 1), the percentage of passaged consensus SNPs that were present as variants in the unpassaged sample is shown on the y- axis. The x-axis shows the name of the passaged descendant identified by sample (1, 27, or 59), host cell type (BO, THP or HRT) and passage number (1, 4 or 5).

We explored the possibility that subconsensus variants support virus adaptation to new cell types by comparing the consensus sequence for each unpassaged parent sample to its passaged descendants. Phylograms generated using the consensus sequences (Figure 1A–D for each of 4 proteins studied) highlight those passaged samples that differed substantially from their unpassaged parents (lower clades), as indicated by their clustering to the NEB strain (upper clades). Figure 2 shows for each sample that clustered away from the unpassaged parent, the percentage of passaged consensus SNPs (single nucleotide polymorphisms) that were present as variants in the unpassaged sample (see Material and Methods for a detailed description of the methods used to rigorously differentiate mutations attributable to sequencing error). The majority of consensus passaged SNPs were present as variants in the respective unpassaged parental samples in all cases except 59.BO1 and 59.BO4, which were impacted by the low variant count in 59 UP (Table S3). Thus results from samples 1 and 27 supports the hypothesis that the mutations present in passage strains were “selected” from a pre-existing pool rather than through *de novo* mutation and subsequent population fixation.

We hypothesized that there are mutation signatures in the genomes of laboratory passaged samples that reflect cell passage adaptation. These mutations are either nonexistent or exist at the subconsensus level in the unpassaged samples, increase in abundance with cell passage, and may eventually reach consensus in highly passaged samples. We identified 186 positions in the BCoV genome where all three unpassaged samples had the same consensus SNP that differed from the consensus SNP found in all eight NEB samples. We defined UP-SNPs and P-SNPs as the consensus SNPs found in the unpassaged parental samples and highly passaged NEB samples respectively, and used the sequence from NEB THP-1 pass 5 (NEB.THP.5) as a highly-passaged representative. Of the 186 P-SNPs, 78%, 24%, and 99% were found to already exist as variants in the unpassaged samples for nasal samples #27, #59 and #1, respectively (using Illumina data), consistent with the results found on a per-sample basis shown in Figure 2.

We further made pair-wise comparisons of early vs. late passaged samples derived from the same nasal sample through the same cell line. For example, 27.THP.1 was compared to 27.THP.5, and 59.THP.1 was compared to 59.THP.5. There were 9 such pairs available for this analysis, 3 cell types (Bomac, THP-1, and HRT-18) for each nasal sample (#27, #59 and #1). Of the 186 positions, 42 positions showed an enrichment of the P-SNPs from the early to the late passaged sample in a majority of the cases (at least 5 of the 9 pairs of the passaged samples). Two of these 42 positions are located on the nsp1 gene, the other 40 are all located on the spike gene (Table S4). The point mutations –base change from UP-SNP to P-SNP – at 17 of the 42 locations are non-synonymous (Table 2).

**Table 2:** Non-synonymous mutations associated with cell passage.

Position	Codon Position	UP-Codon	P-Codon	UP-AA	P-AA	Position-AA (spike)
6461	1 (ts)	TAT	TGT	Y	C	–
23672	1 (ts)	ACG	ATG	T	M	11
***23759***	***1 (ts)***	***ACT***	***ATT***	***T***	***I***	***40***
***24083***	***1 (ts)***	***GGT***	***GAT***	***G***	***D***	***148***
***24145***	***0 (tv)***	***AAT***	***CAT***	***N***	***H***	***169***
***24157***	***0 (tv)***	***AAT***	***CAT***	***N***	***H***	***173***
***24160***	***0 (ts)***	***TCT***	***CCT***	***S***	***P***	***174***
***24382***	***0 (tv)***	***ATG***	***CTG***	***M***	***L***	***248***
***24397***	***0 (tv)***	***AAT***	***TCT***	***N***	***S***	***253***
***24398***	***1 (tv)***	***AAT***	***TCT***	***N***	***S***	***253***
25166	1 (tv)	ACT	AAT	T	N	509
25267	0 (tv)	GCT	TCT	A	S	543
25351	0 (ts)	CAT	TAT	H	Y	571
25458	2 (tv)	TTG	TTT	L	F	606
25559	1 (ts)	ACT	ATA	T	I	640
25945	0 (ts)	TCG	GCG	S	A	769
26357	1 (ts)	GAT	GGT	D	G	906

**Figure 3:**
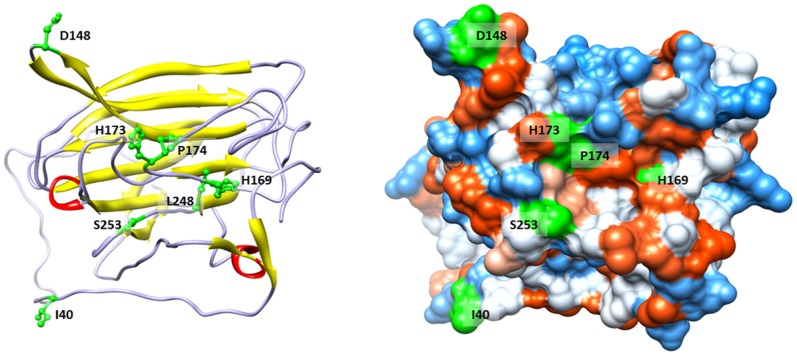
Structural model of the receptor binding domain from the BCoV passaged sample. Ribbons representation (left) and hydrophobic surface (right). Colors of the hydrophobic surface range from blue for the most polar residues to white to orange red for the most hydrophobic residues. Positions of non-synonymous mutations are colored in green (see Table 2).

UP = unpassaged, P = passaged, AA = amino acid, (ts) = transition, (tv) = transversion. Mutations located in N-terminal receptor binding domain in the spike protein (see Figure 3) are in italics.

All 42 passage markers were found as variants in unpassaged samples #1 and #27, but only 15 were identified in unpassaged #59. Since all of sample #1′s passage derivatives rapidly changed with passage to resemble the NEB strain, it was not surprising that all of the “acquired” NEB strain SNPs were present in the initial unpassaged sample. The genetic changes that occurred in the #27 and #59 derived samples showed a less clear pattern since only a subset of samples acquired NEB strain SNPs as the consensus. We checked if the lack of NEB variants in unpassaged sample #59 was an anomaly by comparing the variant pools in its passaged descendants to the NEB SNPs. There indeed was a large increase in NEB strain SNPs existing as variants in the passaged descendants of #59 compared to the parent sample, suggesting these SNPs were either independently acquired after a single cell passage or were undetected by sequencing of the original unpassaged sample. It seems likely that increased numbers of serial passages would result in samples #27 and #59 acquiring more NEB strain SNPs.

### Many of the Passage Associated Mutations can be Mapped to the Surface of the Spike Protein

Of the 42 passage associated mutations, 17 of the mutations were non-synonymous and 16 of these were located in the spike gene (mutation sites are listed in Table S3). All 17 non-synonymous mutations (9 transitions (ts) and 8 transversions (tv)) are shown in Table 2. A structural model of the N-terminal part of the receptor binding domain of the spike protein from the passaged BCoV was constructed using the AS2TS system 5 [24] based on the homology (54% of sequence identity) to the spike protein from a murine coronavirus (PDB template 3r4d_B) [12]. As shown in Figure 3, at least 7 out of 8 of the non-synonymous mutations (see positions colored in green in Table 2) occurred at surface-accessible residues in the receptor binding domain. One of these surface exposed amino-acid mutations, N253S required 2 transversions (AAT – TCT). Only one non-synonymous mutation, M248L (1 transversion), was located in a buried position on the spike protein.

### Haplotype Reconstruction

Given that the 12 nt insert and the majority of the passage associated point mutations were located on the spike protein, we sought to determine whether these passage markers are part of a ‘passaged genotype’, and whether they were linked with the insert on the same haplotype. The open source software, ShoRAH, was used to carry out genotype reconstruction. ShoRAH estimates genetic diversity of a heterogeneous sample from deep sequencing data and has previously been applied to estimating HIV and hepatitis C virus quasispecies [25], [26]. Given any two positions in the genome, ShoRAH returns genotypes found between the two positions based on all reads overlapping the region between the positions.

The possibility that the passage marker mutations were linked to the 12-nt insert on the same genotype was examined. To check for linkage between the SNPs and the insert (at nt position 26374), reads overlapping the two closest passage markers (positions 26361 and 26394) were divided into the “insert-containing” group and the “insert-lacking” group (separately for each sample). For ten of the samples (27.UP, 27.BO.1, 27.THP.1, 27.BO.4, 59.BO.1, 59.THP.1, 59.HRT.1, 59.THP.5, 59.HRT.5, and 1.UP), most of the insert-lacking reads contained the UP-SNPs ‘C’ at 26361 and 26394, and majority of the insert-containing reads showed P-SNPs ‘T’ at these two positions. However, in the case of all of the NEB passaged samples and all passaged samples for nasal sample #1, a different pattern was observed. Here, although the insert-lacking reads occurred at low levels (∼2.5%), they continued to exclusively show the P-SNP variant. The majority genetic population in nasal sample #1 lacked the insert and had the genotype 26361-C, 26394-C while its passaged descendants retained a minority population of insert-lacking genomes that contained the genotype 26361-T, 26394-T (identical to the insert containing dominant genotype). Thus, while there appeared to be two distinct genotypes circulating, it remained unclear whether the insert-lacking passaged variants of nasal sample #1 and NEB originated from a P-variant that lost the insert or rather originated from an insert lacking UP-variant and acquired the P-variant mutations independently.

## Discussion

Deep sequencing of three naturally infected BCoV samples show that each sample differed in the composition of mutant spectra, even within the same herd, and this underscores the importance of including multiple naturally-infected samples in virus evolution studies when possible. Furthermore, identification of a relatively common genetic insert in the BCoV genome that is likely to impact phenotype suggests that this type of in-depth analysis of viral populations may be necessary to fully understand evolutionary mechanisms and interspecies transmission events. Indeed, a similar finding was recently described for foot-and-mouth disease virus (FMDV) where deep Illumina sequencing revealed a genotype present at the subconsensus level that enabled the virus to bind heparin sulfate receptors for adaptation to cell culture [27].

The RT-PCR amplification results suggest that the naturally-infected viruses did not reach sufficiently high titers to allow prolonged serial passage in Bomac cell lines but did replicate relatively efficiently in human macrophage and enteric cell lines. Sequence data indicate that the virus population undergoes rapid change when grown in Bomac cells and this may have made the viral genome difficult to detect with our PCR primers. However, the fact that amplification failed for most regions of the genome simultaneously, even regions that are relatively conserved, indicate that the virus was not replicating efficiently in this environment. Alternatively, BCoV is known to persistently infect cell lines, shedding few viral particles in the supernatant [28] and because RNA was extracted from the supernatant but not the cells, intracellular infections would not have been detected.

Our data indicate that BCoV is able to infect and replicate in human macrophage after exposure to relatively small numbers of virus, and it is possible that this may lead to a more systemic infection due to movement of infected macrophage to the lymph nodes. More mutations were observed in the consensus sequence of the viral genome after passage in macrophage cells, particularly bovine macrophage. This could be a result of increased selective pressure in this cell environment. For example, the production of nitric oxide by macrophage has been shown to increase viral mutation [29].

### Potential Role of Insert in Host Range, Viral Infectivity, and Host Immune Response

Coronavirus infectivity is mediated by the spike protein, which consists of two subunits, the N-terminal S1 subunit and the C-terminal transmembrane S2 subunit. Although the S1 subunit of coronaviruses mediates receptor binding, the S2 subunit mediates fusion of the viral envelope to the host cell membrane [30]. Potential effects of the BCoV insert in the S2 subunit region of the spike gene include increased host cell range via trypsin-independent fusion host cell entry due to the creation of a furin cleavage site [30], and enhanced binding to heparan sulfate on the host cell surface due to the addition of a multibasic region [31]. Although the location of the insert in the S2 subunit may decrease the influence of the insert in heparin sulfate binding, it is interesting to note the presence of other basic residues present in the passaged genotype that may impact binding to heparin sulfate (Figure 3).

**Figure 4:**
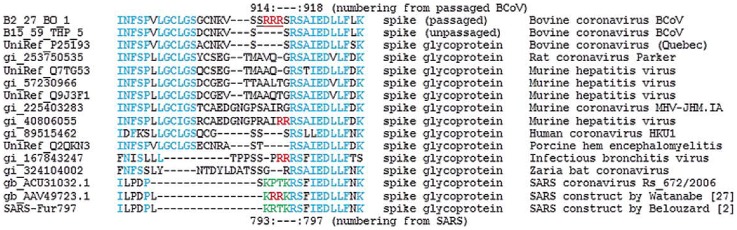
Multiple sequence alignment of BCoV insert region compared with that of other coronaviruses. Sequence region (894–927) including insert SRRR from the passaged BCoV (B2.27.BO.P1) was aligned with other coronavirus spike protein amino acid sequences. The inserted amino acids are underlined; amino acids conserved among coronavirus species are in blue text; RR motifs observed in other sequences are in red. In green is highlighted 793-KPTKR-797 region in SARS-CoV S2 domain described as a furin cleavage site allowed trypsin-independent cell-cell fusion [**23**].

Studies of other coronaviruses show that fusogenic activity of the spike protein may allow for trypsin-independent entry of the virus into a variety of cell types. For example, the addition of a multibasic motif in the S2 subunit of the BCoV spike protein may create a trypsin-independent spike protein activation site as was recently proposed for SARS-CoV [23]. Watanabe et al. [30] generated a SARS-CoV construct with a furin site at the SARS-CoV S2 position (793-KPTKR-797 to 793-KRRKR-797) and have shown SARS-CoV S activation at the cell surface in a trypsin-independent manner. This work highlights the importance of residue R797 in the context of SARS-CoV infection. The position of SARS-CoV residue R797 corresponds to residue 918 in passaged BCoV, and multiple sequence alignments show that this arginine residue is extremely well-conserved across coronaviruses (Figure 4). Interestingly, the two arginines (R794 and R795) from the SARS-CoV construct overlap with arginines from the insert 912-SRRR-915 identified in passaged BCoV.

Although the multibasic insert may enhance binding of BCoV to a diversity of cell types via binding of heparan sulfate on the host cell surface, heparan sulfate binding has been associated with viral attenuation in vivo due to increased clearance rate [32]–[34] and this may provide an explanation for the rarity of the insert genotype in naturally-infected samples. Additionally, coronavirus fixation to heparan sulfate on the surface of macrophages has been associated with increased host inflammatory cytokine response via binding of Toll-like receptor 2 [35]. Other environmental factors such as the presence of trypsin-like enzymes in the bovine airway could also influence selective pressures on the frequency of genotypes lacking the insert [36].

### Comparison of Amino Acid Substitutions to Previous Study Results

Chouljenko et al. (1998) identified amino acid changes in the spike protein gene that differentiate respiratory BCoV (RBCV) strains from enteric BCoV (EBCV) strains, as well as amino acid changes that were “virulence specific”, meaning that they were not detected in extensively lab-passaged strains that had an attenuated phenotype in bovine infections [37]. Our data show 10 of the 13 RBCV-specific changes present in the UP group samples but not the P group samples (Table S5). UP group amino acid changes also correlated with all 7 amino acid changes considered “virulence markers” by Chouljenko et al. (1998). Similarly, 8 of the 10 changes identified in a study by Zhang et al., (1991) as being associated with virulence were present in the UP group but not in the P group [38]. Six of these changes are nonconservative and all of these were reproduced in our data. These results indicate that laboratory passage causes predictable changes in the amino acid sequence, although the rate of change observed in the consensus sequence differs according to cell type and individual sample.

A number of the amino acid changes in the spike protein have been hypothesized to be involved in virulence or tissue tropism of the virus [14]. In particular, amino acid change from Ser in the UP group to Ala in the P group was observed at amino acid 769 which is directly adjacent to the spike protein proteolytic cleavage site. A serine at this site has been hypothesized to be associated with cell fusion [37], and our data indicate loss of the serine is associated with gain of the positively-charged insert in the S2 subunit of the spike protein. Another region immediately upstream of the insert (899–907) has been suggested to be a fusogenic domain by Parker et al. [22]. Interestingly there is a nonconservative amino acid change in this sequence from a nonpolar amino acid, Ala in Parker et al. (1990) and Gly in Chouljenko et al. (1998) and P group, to an acidic amino acid, Asp, in our UP group. This region is composed almost entirely of nonpolar amino acids and the addition of a charged residue may influence function. Another mutation occurred at amino acid 1030 which is part of the heptad repeat, a domain associated with class I fusion proteins and host range [39], in this case a Gly in the UP group was replaced with an Asp in the P group. Ultimately, the role of these mutations in viral phenotype will need to be confirmed using reverse genetics.

Using high throughput sequencing, we tracked the evolution of variant genotypes over several passages through cell lines. The identification of variants through deep sequencing of multiple BCoV samples points to the RNA virus’ relatively high mutation rate. However, the majority of the identified variants were detected in relatively low abundance, leaving open the question of their functional significance. Comparing consensus point mutations with their variant counterparts was used as a way to find variants with greater potential for functional significance. Our results showed that rather than *de novo* mutation being the driver of genetic change at the consensus population level, selection of pre-existing mutations was evident in the majority of cases. Presumably with a sufficiently large population size and extended evolution, novel mutation becomes a bigger factor. The results here illustrate that introduction of novel mutations is a non-trivial process. Moreover, the results indicate the importance of identifying the variants in the population as a potential key piece of information in assessing how a viral isolate would most likely adapt genetically to a new host environment.

## Materials and Methods

### Viruses and Cell Lines

Nebraska (NEB) strain of BCoV was obtained from NVSL (National Veterinary Services Laboratories, Ames, IA) and used as a reference (positive control) for cell culture infections and molecular assays. The stock of NEB strain received from NVSL had been passed >50 times in bovine turbinate and embryonic kidney cells. BCoV strain Mebus was obtained from BEI Resources (Manassas, VA). HRT-18 (human colorectal carcinoma cells) cells (ATCC CRL-11663) were grown on Advanced Dulbecco’s Minimum Essential Medium (D-MEM) (Gibco) with 2 mM L-Glutamine (Gibco), 100 IU/ml Penicillin/100 µg/ml Streptomycin (Gibco) and supplemented with 5% heat-inactivated fetal bovine serum (FBS) (Gibco). EBTr (bovine embryonic tracheal) cells (ATCC CCL-44) and Calu-3 (human lung adenocarcinoma) cells (ATCC HTB-55) were grown on Eagle’s Minimum Essential Medium (EMEM) (Gibco) with 2 mM L-Glutamine, Penicillin/Streptomycin, and supplemented with 10% heat-inactivated FBS. THP-1 (human acute monocytic leukemia) cells (ATCC TIB-202) and Bomac (bovine macrophage [40]; a gift from Dr. Mark Estes, Univ. Texas Medical Branch), were grown on RPMI-1640 Medium (Gibco) with 2 mM L-Glutamine, Penicillin/Streptomycin, and supplemented with 10% heat-inactivated FBS. A549 (human alveolar basal epithelial) cells (obtained from NVSL) and EBL (embryonic bovine lung) cells (ATCC, CCL-185) were grown on Minimum Essential Medium (MEM) (Gibco) with 2 mM L-Glutamine, Penicillin/Streptomycin, and supplemented with 10% heat-inactivated FBS. Above cells were propagated with each optimum culture medium until confluent and maintained in 5% CO2 incubator at 37°C. Cells were detached for passaging using 0.25% Trypsin-EDTA (Gibco), except for THP-1 cells. The THP-1 cells were maintained by either addition of freshly made growth medium or by centrifugation and resuspension of the cells into fresh medium.

### Collection and Identification of Naturally-infected BCoV Samples

The frequency of BCoV infection is not known for healthy cattle therefore a large number of calves were sampled to ensure that at least several positive samples would be obtained. This study was approved by the Institutional Animal Care and Use Committee at Lawrence Livermore National Laboratory (Protocol Number 2009-207). Approximately 200 nasal cultures swabs were collected from calves and analyzed. Sterile polyester swabs were used to collect nasal samples, placed in 2–3 mL of Eagle’s Minimum Essential Medium (Gibco) and transported on ice back to the laboratory. Collected nasal swabs were homogenized in Eagle’s Minimum Essential Medium supplemented with 1% antibiotic-antimycotic solution (Gibco). The sample suspensions were clarified by centrifugation at 2000×g for 30 minutes, filtered through a 0.22 µm filter and aliquots of about 500–1000 µl were stored at −80°C.

The first group of cattle tested was housed individually in outdoor pens on a dairy farm in Davis, CA. It is likely these calves were relatively stressed due to a several day period of very cold and rainy winter weather. Nasal swabs were obtained from 109 animals and 42 of these animals were positive for BCoV infection as determined by RT-PCR assay [41]. Another group of 67 calves were tested during a period of relatively mild winter weather. These calves were part of a herd that included cow-calf pairs that were grazing on pasture. Nasal swabs were obtained from the calves during branding and vaccination procedures. None of these samples were positive for BCoV RNA by RT-PCR assay.

### *In vitro* Passage of Virus

Confluent cell lines in 12-well plates were infected with 200 µl nasal samples diluted in MEM. Virus samples were incubated for 6 hours, inoculums were removed and cells were rinsed twice in sterile PBS. In the case of THP-1 cells, cells were pelleted in between each step. The appropriate media was added to each cell line. Supernatant from infected cells were transferred to fresh cell monolayers every 72 h. NEB strain stock was used as a positive control for infection of each cell line.

### RNA Extraction

A 500 µl sample was homogenized with 500 µl TRIzol reagent (Invitrogen) following manufacturer’s protocol except that incubations were performed at 30°C instead of room temperature.

### Primer Design

Primers were designed to amplify eight regions of 1.5 to 2.5 kb and amplified regions overlapped to allow the genotypes to be reconstructed. Primers were designed to be as sensitive to target strain variants as possible, while still being specific enough to not cross-react with non-targets. In all, 24 primer sets (3 sets for each genome region) were tested to select the two best performing primer sets for amplification of each region (Table S1).

### Reverse Transcriptase (RT) Polymerase Chain Reaction (PCR)

RNA was converted to cDNA using random hexamers and the SuperScript III First-Strand Synthesis System for RT-PCR (Invitrogen) according to manufacturer’s instructions. In cases where viral titer appeared to be low, 10 µl RNA was added to the RT mixture. The PCR primers and conditions used to detect BCoV RNA present in nasal samples are described in Cho et al., 2001 [41]. High fidelity Phusion polymerase (New England BioLabs) was used to amplify regions of the BCoV genome for sequencing following manufacturer’s instructions, using 50 µl reactions with 5 µl of cDNA template. The primer sets used for genome amplification are listed in Table S1. PCR conditions consisted of 94°C for 2 min, followed by 40 cycles of 98°C for 10 s, 60°C for 20 s, and 72°C for 1 min 20 s.

### Sequencing of a 12 kb Region of the Genome

Each sample was amplified with each primer set and products were pooled, purified using the QIAquick PCR Purification kit (Qiagen), and quantified. Amplicons were submitted for 454 sequencing at DNA Sequencing Center at Brigham Young University and a subset of amplicons were sequenced on an Illumina GA IIx sequencer at Eureka Genomics Hercules, CA. The raw sequencer reads generated from the experiments and the reads aligned to the reference sequence is available at ftp://gdo-bioinformatics.ucllnl.org/pub/bcv1.

### Taqman PCR

Taqman assays (qRT-PCR) were designed to detect a region of the virus polymerase gene as well as a region of the spike gene that was found to be the site of 12 nucleotide (nt) insert for some samples. RNA was extracted and reverse transcribed as described above. Taqman assay was performed on a 5 µl aliquot of cDNA in 50 µl reaction mix using the 7900 HT Sequence Detection System (Applied Biosystems) and Taqman Universal PCR Master Mix (Applied Biosystems) for amplification of the viral cDNA. Plasmids cloned with inserts from amplification of a larger region of the polymerase gene or spike insert region were used to generate the standard curve for quantitation. Taqman primers and probe for amplification of the polymerase gene were (5′ to 3′): Fwd- CCATGTGTCATGCATTGGATT, Rvs- CACCGATCATCCTGACAATCA, Probe- CCGTGTTAGGATGGTATGGCATACTCCAGTG. Taqman primers and probe for amplification of the spike insert region were (5′ to 3′): Fwd- GGTTGTTTAGGAAGCGGTTG, Rvs- AGCCTCAACGAAACCGACAT, Probe- CCGGCGTAGAAGTAGATCTGCTATAGAGGATTT. Cycling parameters were 50°C for 2 min, 95°C for 10 min, 45 cycles of 95°C for 15 sec and 60°C for 1 min.

### Control for Analysis of PCR and Sequencing Error Rates

To determine the error rate of the PCR amplification and sequencing processes, a sequencing control plasmid was constructed by inserting a 1 kb fragment of the BCoV polymerase gene into a sequencing plasmid using an Invitrogen TOPO® Cloning Kit for Sequencing. The plasmid region was then Sanger sequenced to determine the sequence of the insert prior to PCR amplification and 454 or Illumina sequencing. The plasmid insert was amplified using the same protocol as the viral samples and submitted for 454 and Illumina sequencing along with the viral PCR products.

### Viral Sequence Assembly

The sequencing control was used to model error by measuring observed miscall rates of non-consensus bases. Sample consensus sequences were iteratively constructed by first mapping to a closely related reference sequence (Genbank Identifier 15081544), constructing a new consensus sequence, mapping the reads to the new consensus sequence as the reference (in order to increase the number of mapped reads and reduce the potential for alignment error), and repeating the process until the consensus sequence converged to a single value.

The following error filtering rules were used to preclude a read that has been aligned to the consensus sequence from contributing to a base call at a given genome position due to likelihood of error: an indel (in the aligned read) cannot be present within five bases of the query base, the position within the read must be at least five bases away from the ends of the read (to further avoid misalignment); any variant call must be observed in reads that were sequenced from both strands. To further avoid miscalls from misalignment, the adjacent bases in the read (+/−1) must agree with the consensus sequence thus filtering out adjacent variant mutations. These rules eliminated a large percentage of the erroneous variant calls in the control sequences. Thus, rather than relying on a uniform observed error rate covering all positions in the control, we used the average per position, per base call error rate restricted to the cases where variant base calls were identified. The reasoning was that an alternative approach would lead to an artificial lowering of the error rate that includes cases where no variant base calls were observed. This led to an observed per genome position, per base call conditional error rate of 0.001, which reflects the average percentage of reads reporting an erroneous sub-consensus base call at each position in the genome.

The error rate was conditioned on the case when a non-consensus base call is made, rather the error rate averaged over all base calls including those where no-subconsensus mutation is attempted due to the read mapping and quality control exclusion rules and subsequent lack of observed variation. Since this is at least an order of magnitude greater than the mutation frequency expected of coronaviruses it is necessary to observe the sub-consensus mutation in multiple sequencer reads to ensure that a mutation occurs with a high enough frequency in the sample to be unlikely attributable to sequencing error.

To determine the minimum number of reads needed to support the presence of a sub-consensus mutation, the Binomial significance test described by Eriksson et al. (2008) was applied. This approach assumes a binomial error distribution so that for a given sequencer coverage amount, the number of reads containing sub-consensus mutations that would be expected from the assumed sequencing error rate can be calculated. When a mutation occurs in a number of reads that exceeds this amount for a P-value of 0.01 (and applying a Bonferonni correction), the mutation is inferred to be unlikely attributable to sequencing error. The detection sensitivity is therefore coverage and sequencer error dependent. For coverage 1,000× the mutation would be required to occur in 5 reads, which constitute 0.5 percent of the total with at least two reads occurring on opposite strands.

Illumina sequencing was carried out on four samples (Table S3) using Illumina paired-end technology where read pairs of length 112 bp were created with an overlapping region of approximately 80 bp. Although the paired-end technology was originally intended to create read pairs with gaps of known size when the templates are cut to lengths much longer than the read length (e.g. 500 bp templates and 100 bp reads). The known gap size facilitates post-sequencing alignment that is particularly useful for more complex non-viral genomes. When the templates used are closer to the read length the paired-end technology creates read pairs with significant regions of overlap.

These overlapping read pairs essentially provide another mechanism of error checking, as each read pair came from the same template and therefore should be perfectly complementary. Any disagreement between the read pairs would be due to sequencing error. This disagreement between overlapping read pairs was used to correlate mismatch rates and the sequencer generated quality scores. A cutoff of Q30 was selected as the minimum base call quality score, which limits the mismatch error rate, while maintaining high amounts of sequencer coverage. Only base calls supported by both read pairs with the minimum quality score were used to infer an affective erorr rate in the control sequence, which was found to be 0.0005. This rate is the maximal percentage of reads that were observed to disagree at any position in the control. As with the 454 data, this error rate was used as input to the Binomial significance test to determine the minimum number of reads needed to separate sequencing error from true genetic variation.

Open source software, ShoRAH, was used to carry out genotype reconstruction [25], [26].

454 Pyrosequencing data were obtained for 30 BCoV samples: 7, 7, 8 and 8 samples of nasal samples #27, #59, #1, and the NEB strain, respectively. Across all 30 samples, 12,159 unique bases were sequenced, covering nsp1, nsp2, nsp3 and spike proteins between positions 2647 and 27809 on a reference genome, GenBank accession number NC_003045. Average coverage at the four protein regions are 531×, 2535×, 588× and 623×. The standard deviations of coverage at these four protein regions are 210×, 587×, 108× and 270×. Hence, coverage at the nsp2 genome was both highest and most variable among the four proteins. Four of the 30 samples were also sequenced using Illumina to obtain deeper coverage and clarify differences with the 454 samples. Table S3 summarizes the counts of variants detected and the average per base read coverage in each sample, and includes the number of genome positions with 50×coverage or greater, which was used as the minimum coverage cutoff for consensus base calls. Primer regions were excluded to avoid false positive variant calls from non-specific primer binding. An average of 10.7 kb consensus sequence was recovered.

## Supporting Information

Figure S1 **Average coverage per sample (y-axis) versus count of rare variants detected in each sample, with a linear fit.**
(DOCX)

Table S1 **Primers used to amplify regions of the BCoV genome.**
(DOCX)

Table S2 **Amplification of BCoV RNA from NVSL by Taqman using Polymerase and Insert Primers.**
(DOCX)

Table S3 **Summary deep sequencing data.**
(DOCX)

Table S4 **Putative genetic markers of cell culture adaptation.**
(DOCX)

Table S5 **Comparison of amino acid changes induced during passage.** Laboratory-adapted strains (Mebus and L9) and low passage strains or unpassaged strains
(DOCX)
