# Supplementary material for: The Role of Viral Population Diversity in Adaptation of Bovine Coronavirus to New Host Environments
Source: PLoS One. 2013 Jan 7;8(1):e52752. doi: 10.1371/journal.pone.0052752 (PMC3538757; doi:10.1371/journal.pone.0052752)
Supplement: Figure S1 — Average coverage per sample (y-axis) versus count of rare variants detected in each sample, with a linear fit. (DOCX) [file pone.0052752.s001.docx]

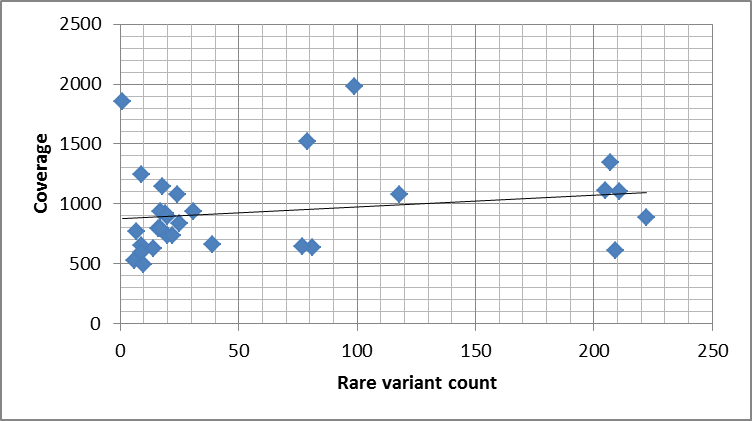


**Suppl. Figure 1. Average coverage per sample (y-axis) versus count of rare variants detected in each sample, with a linear fit.**
