# Supplementary material for: The Role of Viral Population Diversity in Adaptation of Bovine Coronavirus to New Host Environments
Source: PLoS One. 2013 Jan 7;8(1):e52752. doi: 10.1371/journal.pone.0052752 (PMC3538757; doi:10.1371/journal.pone.0052752)
Supplement: Table S2 — Amplification of BCoV RNA from NVSL by Taqman using Polymerase and Insert Primers. (DOCX) [file pone.0052752.s003.docx]

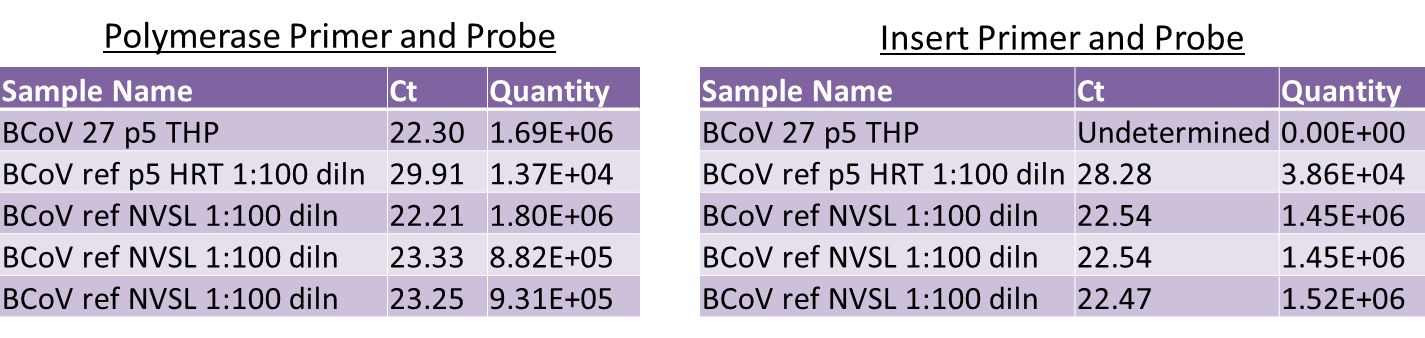


**Supplementary Table 2**. **Amplification of BCoV RNA from NVSL by Taqman using Polymerase and Insert Primers.** The reverse transcription reaction was performed with ~250 ng BCoV RNA shipped from National Veterinary Services. Taqman amplification was performed in triplicate using a 100-fold dilution of the cDNA synthesized. Data from sample 27 passage 5 in THP-1 cells and Nebraska reference strain, passage 5 in HRT-18 cells are shown for comparison. In each case, a Taqman assay was performed as described in the Materials and Methods section using primers and probe for the BCoV polymerase to determine the approximate copy number of BCoV genomes present in each sample, and a Taqman assay was performed to determine the approximate number of genomes with insert. Quantities for insert based on standard curve generated for the polymerase control plasmid.
