# Supplementary material for: The Role of Viral Population Diversity in Adaptation of Bovine Coronavirus to New Host Environments
Source: PLoS One. 2013 Jan 7;8(1):e52752. doi: 10.1371/journal.pone.0052752 (PMC3538757; doi:10.1371/journal.pone.0052752)
Supplement: Table S3 — Summary deep sequencing data. (DOCX) [file pone.0052752.s004.docx]

| **Sample** | **Subconsensus variants** | **Average coverage** | **Base calls** | **Median subconsensus frequency** |
| --- | --- | --- | --- | --- |
| B1,27,na,UP | 81 | 635 | 12287 | 0.03597 |
| B2,27,BO,1 | 77 | 642 | 12299 | 0.02827 |
| B3,27,THP,1 | 9 | 656 | 12317 | 0.04787 |
| B4,27,HRT,1 | 6 | 526 | 12316 | 0.04037 |
| B6,27,BO,4 | 79 | 1522 | 6719 | 0.33277 |
| B7,27,THP,5 | 9 | 590 | 12293 | 0.03907 |
| B8,27,HRT,5 | 20 | 736 | 12300 | 0.02927 |
| B9,59,na,UP | 10 | 490 | 12292 | 0.04717 |
| B10,59,BO,1 | 209 | 612 | 12317 | 0.44487 |
| B11,59,THP,1 | 211 | 1103 | 12316 | 0.18467 |
| B12,59,HRT,1 | 118 | 1080 | 12318 | 0.01957 |
| B13,59,EBL,1 | 99 | 1983 | 6270 | 0.01997 |
| B15,59,THP,5 | 222 | 889 | 12299 | 0.25427 |
| B16,59,HRT,5 | 205 | 1112 | 12300 | 0.23687 |
| B17,1,na,UP | 207 | 1342 | 12167 | 0.05147 |
| B18,1,BO,1 | 9 | 1242 | 10387 | 0.03797 |
| B19,1,THP,1 | 1 | 1853 | 2295 | 0.05177 |
| B20,1,HRT,1 | 19 | 916 | 12331 | 0.03377 |
| B21,1,EBL,1 | 31 | 933 | 7015 | 0.14497 |
| B22,1,BO,4 | 7 | 770 | 10664 | 0.24217 |
| B23,1,THP,5 | 22 | 738 | 10802 | 0.12417 |
| B24,1,HRT,5 | 17 | 937 | 12332 | 0.03297 |
| B25,NEB,BO,1 | 18 | 1144 | 8793 | 0.04057 |
| B26, NEB,THP,1 | 24 | 1080 | 8793 | 0.01797 |
| B27, NEB,HRT,1 | 20 | 895 | 10819 | 0.03107 |
| B28, NEB,A549,1 | 16 | 795 | 10842 | 0.04517 |
| B29, NEB,EBL,1 | 25 | 837 | 10839 | 0.03007 |
| B30, NEB,BO,4 | 39 | 662 | 10387 | 0.12297 |
| B31, NEB,THP,5 | 14 | 630 | 12332 | 0.02847 |
| B32, NEB,HRT,5 | 17 | 796 | 12331 | 0.02997 |
| **Illumina Data** | | | | |
| B01,27,UP,NA | 226 | 46339 | 12158 | 0.005066121 |
| B09,59,UP,NA | 122 | 54933 | 12161 | 0.004966121 |
| B17,1,UP,NA | 260 | 10420 | 12164 | 0.056566121 |
| B14,59,BO,4 | 77 | 39913 | 6905 | 0.005005346 |

Supplementary Table 3. Summary deep sequencing data. Columns 1-5 show sample (sequence identifier number (B#), nasal sample #, cell line, and passage #), subconsensus variant counts, average sequencing coverage, number of positions in the genome with consensus base calls, and median frequency of subconsensus variants, respectively.
