# Supplementary material for: The Role of Viral Population Diversity in Adaptation of Bovine Coronavirus to New Host Environments
Source: PLoS One. 2013 Jan 7;8(1):e52752. doi: 10.1371/journal.pone.0052752 (PMC3538757; doi:10.1371/journal.pone.0052752)
Supplement: Table S4 — Putative genetic markers of cell culture adaptation. (DOCX) [file pone.0052752.s005.docx]

| Gene | Base positions in Ref genome* |
| --- | --- |
| Nsp1 | 6461^†^, 6471 |
| Nsp3 | none |
| Nsp14 | none |
| Spike | 23672^†^, 23709, 23733, 23759^†^, 24083^†^, 24145^†^, 24157^†^, 24160^†^, 24270, 24382^†^, 24397^†^, 24398^†^,  24828, 25011, 25166^†^, 25173, 25248, 25254, 25267^†^, 25281, 25302, 25351^†^, 25365, 25452,  25458^†^, 25461, 25559^†^, 25569, 25668, 25698, 25761, 25770, 25785, 25821, 25827, 25945^†^,  26097, 26357^†^, 26361, 26394, |

Supplementary Table 4. Putative genetic markers of cell culture adaptation.

*Numbers from accession # NC_003045

^†^ Positions where UP-SNP to P-SNP base substitutions led to amino acid changes
