# Supplementary material for: The Role of Viral Population Diversity in Adaptation of Bovine Coronavirus to New Host Environments
Source: PLoS One. 2013 Jan 7;8(1):e52752. doi: 10.1371/journal.pone.0052752 (PMC3538757; doi:10.1371/journal.pone.0052752)
Supplement: Table S5 — Comparison of amino acid changes induced during passage. Laboratory-adapted strains (Mebus and L9) and low passage strains or unpassaged strains (DOCX) [file pone.0052752.s006.docx]

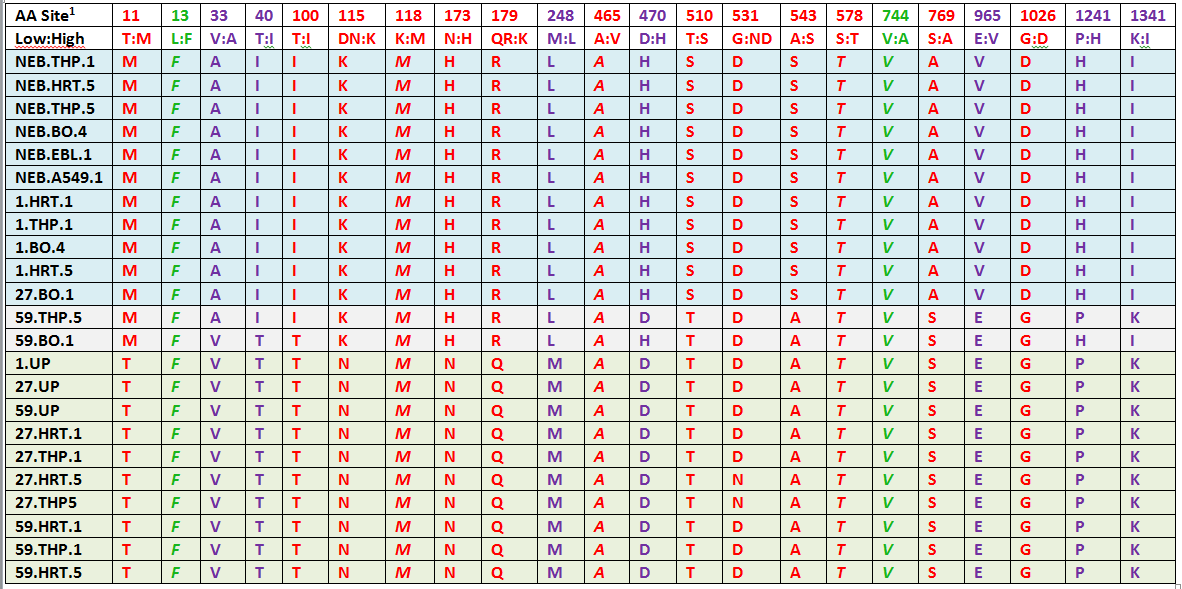


**Supplementary Table 5. Comparison of amino acid changes induced during passage. Laboratory-adapted strains (Mebus and L9) and low passage strains or unpassaged strains** from Chouljenko et al., 1998 are shown in second row (low passage: high passage) and compared to data from this study (shown in the lower 23 rows of the table). Amino acid changes associated with high passaged, lab-adapted strains with attenuated phenotype versus low passage strains from clinical isolates are shown in purple text. Amino acid changes associated with a respiratory BCoV samples in the Chouljenko study are shown in red text, and amino acids associated with enteric samples are shown in green. Amino acids that did not change in this study as was described in the Chouljenko paper are shown in italics. Samples from this study in blue-shaded cells are from the P Group, those in green-shaded cells are in the UP Group, and samples with grey-shaded cells are located between UP and P in the phylogram (Figure 1). Samples with incomplete sequence data for the spike gene were not included in the analysis. 1: Amino acid site in spike protein gene.
