# Supplementary material for: The Role of Viral Population Diversity in Adaptation of Bovine Coronavirus to New Host Environments
Source: PLoS One. 2013 Jan 7;8(1):e52752. doi: 10.1371/journal.pone.0052752 (PMC3538757; doi:10.1371/journal.pone.0052752)
Supplement: Supplementary file 6 [file pone.0052752.s002.docx]

Identifier	Sequence (5' to 3')
A_FOR_2_2616_BCV	TGTGGTTATTCTGAACCACCTAAAGTTGCA
A_FOR_3_2652_BCV	ATTTGCATTGTGGATAATGTTTATATGGCC
A_REV_2_4797_BCV	CGGGACACTCAAAATACTTAACGG
A_REV_3_4854_BCV	AACTACCCTGCTGTACATAACCAAA
B_FOR_1_4600_BCV*	TAAGCAATGACGTTGCATTTGTTTCG
B_FOR_2_4643_BCV	GGATGTTTTATCCTTAAGACATGATATAGC
B_FOR_3_4674_BCV	CTTGATGATGATGCACGAACCTTT
B_REV_1_6519_BCV*	ACTTACGTATTGTAGGTACGTTAACTGC
B_REV_2_6542_BCV	AAGAGTCATACCAAATTTTATAAACTTACG
B_REV_3_6580_BCV	ATCTCTCTTAAATTTAACAAATCAATTGGT
C_FOR_2_6344_BCV	AAAACCATTTAAGGTTGAAGATAGTGTCAT
C_FOR_3_6390_BCV	AGTGAAATCAAATATGTTAAGAGTTTGTCT
C_REV_2_8570_BCV	AAGCTGAAAATCTGATTTGTGTACTGTGTA
C_REV_3_8625_BCV	CGCTAACATCTCTAATAACACCATTATCTA
D_FOR_1_10700_BCV	AGCTTCTATGACTGGTGTGTCTTTGGA
D_FOR_2_10755_BCV	AAGAATGGTTTTCAAGGACGTCAGAT
D_FOR_3_10765_BCV**	TTCAAGGACGTCAGATTATGGGTAGTT
D_REV_1_11783_BCV	AACATGCAAATGTTGCAAGCAATTAAGCAA
D_REV_2_11836_BCV	AGTATTTCATTGTGCAAAGTGCTACAATA
D_REV_3_11743_BCV**	GCACATTTAACATCAGTCAATTTTGATTGA
E_FOR_2_17743_BCV	TGCAGAAACAGCGCATTCTGTAAATGTTAA
E_FOR_3_17764_BCV	AAATGTTAATCGCTTCAATGTTGCTATTAC
E_REV_2_19532_BCV	CACCAGCCTGTCCTGTATAATGACCAGT
E_REV_3_19545_BCV	GCACAAGGCATTTCACCAGCCTGTC
F_FOR_2_23542_BCV	CTTTTGGGTGTTGCGGTCATAATTATTGTA
F_FOR_3_23588_BCV	TTATGGTGGATAATGGTACTAGGCTGCAT
F_REV_2_25077_BCV	AACAATGTTGTGCATAAACAACATCATGAT
F_REV_3_25124_BCV	CAAAGACCCATCCAATTTACACGGACAGA
G_FOR_2_24931_BCV	ATAATTTACCTGCTGCTAATGTTTCTGTTA
G_FOR_3_24969_BCV	AATCCTTCTACTTGGAATAGGAGATTTGG
G_REV_2_26452_BCV	CAATTATTATAAGCCTCAACGAAACCGACA
G_REV_3_26437_BCV	TCAACGAAACCGACATCAGATAACTTT
H_FOR_1_26204_BCV	CACAGAAGTAAATGAACTACTTGACACTAC
H_FOR_2_26222_BCV	ACTTGACACTACACAGTTGCAAGTAGCTAA
H_REV_1_27837_BCV	TGTGGTAGCTATTATAATATGCTCGACCT
H_REV_2_27852_BCV	CTAAACAGCAGGCATTGTGGTAGCTATTAT

Supplementary Table 1. Primers used to amplify regions of BCoV genome.

*Alternate primer set used to amplify 27 THP pass 5.

** Alternate primer set used to amplify 59 Bomac pass 4, and NEB strain Bomac pass 4
